# Investigation of Nonmotor Symptoms in First-Degree Relatives of Patients with Different Clinical Types of Parkinson's Disease

**DOI:** 10.1155/2019/1654161

**Published:** 2019-01-02

**Authors:** Jiang-bing Liu, Jun-ling Leng, Ying-ge Wang, Yu Zhang, Tie-yu Tang, Li-hong Tao, Xin-jiang Zhang, Chun-feng Liu

**Affiliations:** ^1^Department of Neurology, The Affiliated Hospital of Yangzhou University, Yangzhou University, Jiangsu, China; ^2^Emergency Department, The Affiliated Hospital of Yangzhou University, Yangzhou University, Jiangsu, China; ^3^Department of Neurology and Suzhou Clinical Research Center of Neurological Disease, The Second Affiliated Hospital of Soochow University, Suzhou, Jiangsu, China

## Abstract

**Background:**

Nonmotor symptoms (NMS) are prodromal characteristics of Parkinson's disease (PD). The first-degree relatives (FDR) of PD patients had a higher risk of PD and also had more NMS.

**Objective:**

To delineate NMS in FDR of patients with different clinical types of PD.

**Methods:**

A total of 98 PD probands were recruited; 256 siblings of them were enrolled in the FDR group. Various scales were used to assess NMS, including depression, anxiety, cognitive impairment, insomnia, constipation, excessive daytime sleepiness, rapid eye movement sleep behavior disorder (RBD), and restless legs syndrome (RLS). The incidences of NMS were further compared between the FDR groups of PD with different types.

**Results:**

The FDR of early-onset PD (EOP) showed a higher incidence of moderate to severe depression (OR = 4.08; 95% CI: 1.12–14.92; *P*=0.033), anxiety (OR = 4.22; 95% CI: 1.87–9.52; *P*=0.001), and excessive daytime sleepiness (OR = 3.40; 95% CI: 1.00–11.48; *P*=0.049) than the FDR of late-onset PD (LOP). It was also found that RBD (OR = 11.65; 95% CI: 3.82–35.54; *P* < 0.001), constipation (OR = 4.94; 95% CI: 1.85–13.21; *P*=0.001), sleep disorders (OR = 4.51; 95% CI: 1.73–11.78; *P*=0.002), cognitive impairment (OR = 3.55; 95% CI: 1.62–7.77; *P*=0.002), and anxiety (OR = 2.49; 95% CI: 1.32–4.71; *P*=0.005) were more frequent in FDR of tremor-dominant PD (TDP) than in FDR of non-tremor-dominant PD (NTDP).

**Conclusions:**

The siblings of patients with EOP and TDP have more NMS, presuming that they have a higher risk in the PD prodromal stage. Whether they have a greater possibility to progress into PD requires further investigation.

## 1. Introduction

Parkinson's disease (PD) is the second most prevalent of all the neurodegenerative diseases [1]. It has been considered a sporadic disease for a long period, and only 15% of patients have family history [2]. However, multiple family aggregation studies supported that the relatives of PD, especially the siblings of patients, had a higher risk of PD than the relatives of non-PD patients [3, 4]. Also, the first-degree relatives (FDR) of patients with PD are generally at the increased risk of nonmotor symptoms (NMS), such as anxiety, depression, and dementia [5, 6].

In recent years, people have come to realize that the appearance of motor symptoms does not represent the time when neurodegenerative changes occur. Neurodegeneration occurs several years or even decades before the onset of motor symptoms. Motor symptoms also do not appear immediately for the degeneration in the substantia nigra (SN). Pathological studies have shown a 40%–60% threshold of dopamine neuron loss in the SN pars compacta (SNc) and 60%–70% striatal dopaminergic reduction before the appearance of typical motor symptoms meeting PD diagnostic criteria [7]. Pathological studies also demonstrated that the accumulation of alpha-synuclein occurred first outside the SN, possibly even in the periphery, providing a theoretical basis for the emergence of the prodromal PD [8–10]. An International Parkinson Disease and Movement Disorder Society (MDS) [11] task force proposed the terminology for the early stage of PD, that is, prodromal PD, in which affected subjects might have some NMS and/or subtle motor signs, but no typical motor symptoms meeting the diagnostic criteria for PD are observed.

Our previous study showed that the siblings had more NMS, such as depression, anxiety, and RBD, compared with the normal controls [12]. It is believed that the FDR represented by siblings of patients with PD are prone to have PD prodromal symptoms. Epidemiological studies supported that the early-onset PD (EOP) had more obvious family aggregation [13, 14]. It is speculated that genetic factors and early living environment factors are crucial in the pathogenesis of EOP. In addition, studies have confirmed that patients with non-tremor-dominant PD (NTDP) had a higher risk of dementia compared with tremor-dominant PD (TDP) [15, 16]. So, this study was designed to ask whether FDR of EOP patients differed from LOP patients with respect to the prevalence of NMS. We also asked whether NMS differed in FDR of TDP patients, compared to NTDP patients.

## 2. Materials and Methods

### 2.1. Participants

**Figure 1:**
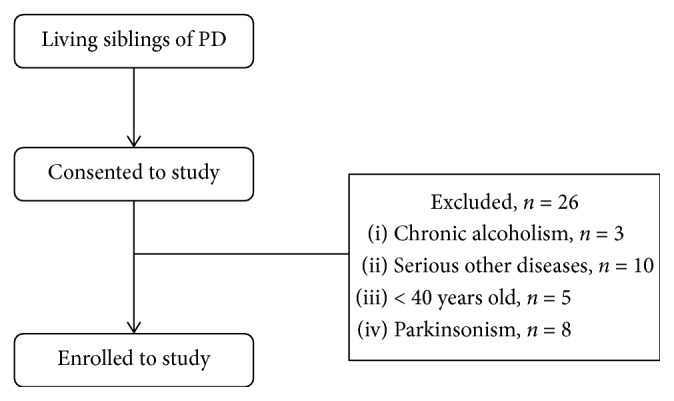
Flow diagram of subject participants.

The PD probands admitted to the Department of Neurology in the Affiliated Hospital of Yangzhou University were recruited for the study between January 2015 and August 2017. The clinical diagnosis of idiopathic PD was determined based on UK Parkinson's disease Brain Bank criteria [17]. Only siblings that shared the same parents were included, and the children and parents of PD probands were excluded to ensure the same early living environment and the same genetic background from parents. Of living, 348 siblings of 98 PD patients invited to take part in this study, 282 consented, 66 refused to participate, and 26 individuals were excluded (Figure 1). The age is the risk factor for PD, and younger brothers and sisters of patients with PD are relatively too young. As a result, they may not have reached the age of symptom onset. Consequently, the siblings who were younger than 40 years old were excluded. At the same time, other interferences that might result in cognitive impairment and emotional disorders, including chronic alcoholism, severe congenital disability, or serious other diseases, were also excluded. FDR subjects who were found to be the patients with parkinsonism were also excluded from this survey. This study was approved by the ethics committee of the Affiliated Hospital of Yangzhou University, and informed consent was obtained from all participants.

### 2.2. Methods

The baseline data of all the subjects were recorded, including age, gender, years of education, vascular risk factors, and lifestyle habits. The vascular risk factors mainly included diabetes, hypertension, hyperlipidemia, cardiac insufficiency, coronary disease, arrhythmia, cerebral infarction, transient ischemic attack, and cerebral hemorrhage. The lifestyle habits of all the participants included drinking coffee (more than three cups per week), drinking tea (more than six cups per week), and smoking.

The age of onset and motor phenotypes of patients with PD were recorded. The age of onset ≤50 years was considered to be early-onset PD (EOP), and the age of onset >50 years was late-onset PD (LOP). PD probands were classified into two motor phenotypes, TDP and NTDP. Following the classification method [18], PD patients were classified into three motor phenotypes, TDP, postural instability/gait difficulty patients (PIGD), and indeterminate patients, based on their MDS-unified Parkinson's disease rating scale (UPDRS) motor score. NTDP includes both PIGD and indeterminate patients in this study.

All the subjects were interviewed by trained investigators and underwent examinations. Restless legs syndrome (RLS) was diagnosed by professional neurologists in accordance with the RLS diagnostic criteria proposed by the International RLS Research Group in 2014 [19]. The various NMS of all the subjects were assessed by researchers who had received questionnaire training.

### 2.3. Questionnaires

Validated questionnaires were used to assess various NMS across (1) rapid eye movement sleep behavior disorder (RBD screening questionnaire (RBDSQ)); (2) excessive daytime sleepiness (Epworth Sleepiness Scale); (3) constipation (constipation scoring system); (4) sleep disorder (Pittsburgh Sleep Quality Index (PSOI)); (5) cognitive impairment (Montreal cognitive assessment (MoCA)); (6) depression (Beck Depression Inventory-II (BDI-II)); (7) anxiety (Zung Self-Rating Anxiety Scale (SAS)). Details of the questionnaires and thresholds for positive symptoms were previously published [12].

### 2.4. Statistical Analysis

Continuous variables were expressed as mean ± standard deviation (SD) and analyzed using *t* tests if they were normally distributed. Continuous variables that were not normally distributed were analyzed using the Kruskal–Wallis nonparametric test. Categorical variables were expressed as frequency (%) and compared using the chi-square or Fisher's exact test. Multivariate binary logistic regression models were used to compare the frequency of various NMS in different subgroups of FDR. Odds ratios (ORs) and 95% confidence intervals (95% CI) were used to evaluate the risks of NMS after adjusting for important confounders. Significant variables in univariate analysis and clinically significant parameters were involved in the multivariable models. We ultimately included age, gender, years of education, smoking, and caffeine intake in adjusted models. All *P* values were two-tailed, and the difference with *P* < 0.05 was considered statistically significant.

## 3. Results

### 3.1. Clinical Data

During the study, a total of 98 PD probands were enrolled, including 11 with EOP (11.2%) with an average age of 45.9 ± 4.9 years (minimum age: 39 years) and 87 with LOP (88.8%) with an average age of 65.8 ± 7.7 years. Further, 45 had TDP (45.9%) and 53 had NTDP (54.1%). 256 siblings of PD were enrolled in the FDR group, including 36 siblings of patients with EOP and 220 of those with LOP. At the same time, these included 128 siblings of patients with TDP and 128 of those with NTDP.

### 3.2. Analyses Comparing the Siblings of EOP vs. LOP

The FDR of EOP have longer years of education than FDR of LOP (8.7 ± 3.6 vs. 7.2 ± 4.2; *P*=0.04). No significant differences in age, gender, smoking rate, habit of drinking tea or coffee, or vascular risk factors were found between the FDR groups of EOP and LOP in this survey (Table 1).

We found that moderate to severe depression (OR = 4.08; 95% CI: 1.12–14.92; *P*=0.033) and moderate to severe anxiety (OR = 9.37; 95% CI: 2.86–30.67; *P* < 0.001) were significantly more frequent in FDR of patients with EOP than in FDR of LOP. In addition, FDR of EOP have a higher frequency of excessive daytime sleepiness (OR = 3.40; 95% CI: 1.00–11.48; *P*=0.049). No significant differences in the incidences of RLS, RBD, constipation, sleep disorders, and cognitive impairment (including possible MCI and dementia), mild depression, or mild anxiety were found between the two FDR groups (Table 2).

### 3.3. Analyses Comparing the Siblings of TDP vs. NTDP

FDR of patients with TDP were younger than FDR of patients with NTDP (64.6 ± 8.4 vs. 67.4 ± 10.5; *P*=0.02). No significant differences in gender, years of education, smoking rate, a habit of drinking tea or coffee, or the vascular risk factors were found between the two groups (Table 3).

It was found that RBD (OR = 11.65, 95% CI: 3.82–35.54; *P* < 0.001), constipation (OR = 4.94; 95% CI: 1.85–13.21; *P*=0.001), sleep disorders (OR = 4.51; 95% CI: 1.73–11.78, *P*=0.002), cognitive impairment (OR = 3.55; 95% CI: 1.62–7.77; *P*=0.002), and anxiety (OR = 2.49; 95% CI: 1.32–4.71; *P*=0.005) were significantly more frequent in FDR of patients with TDP than in FDR of NTDP. The incidences of possible MCI (OR = 3.19; 95% CI: 1.51–6.74; *P*=0.002) and mild anxiety(OR = 2.52; 95% CI: 1.24–5.15; *P*=0.011) in the FDR of TDP were also significantly higher than those in FDR of NTDP, while there were no differences in the possible dementia (OR = 2.64; 95% CI: 0.53–13.08; *P*=0.235) and moderate to severe anxiety (OR = 1.79; 95% CI: 0.62–5.13; *P*=0.279) between the two groups. No significant differences in the incidences of RLS, excessive daytime sleepiness, or different degrees of depression were observed between the two groups (Table 4).

## 4. Discussion

This study explored the NMS of FDR of patients with PD based on the representative of siblings of patients with PD. A previous study showed that the incidences of depression, anxiety, and RBD were significantly higher in FDR of patients with PD than in the controls [12]. Further stratification by the age of onset showed that the moderate to severe depression and anxiety were more pronounced in siblings of patients with EOP compared with siblings of patients with LOP. Also, excessive daytime sleepiness in siblings of EOP showed a higher frequency. At the same time, stratification of motor phenotypes of probands revealed that the siblings of patients with TDP had a higher frequency of various NMS, including RBD, constipation, sleep disorders, possible MCI, and mild anxiety.

Familial-aggregation research results revealed that the relatives of patients with PD had a higher risk of PD compared with the population without PD family history. In addition, it was also found that the risk of PD was higher in relatives of patients with EOP than in relatives of patients with LOP [14]. Arabia et al. [5] analyzed the medical records of 1000 FDR of PD probands and found an increased risk of anxiety disorders and depressive disorders in FDR of PD probands compared with the controls. They also found an increase in the risk of depression in relatives of patients with age at onset <66 years (hazard ratio (HR) = 1.95). The present study found that the incidences of moderate to severe depression and anxiety were higher in the siblings of EOP than in the siblings of LOP, which was partly consistent with the previous findings. In a population-based study [6], the cognition impairment information of FDR of patients with PD was obtained using the telephone and medical records linkage system. They found that the risk of cognitive impairment or dementia increased in FDR of patients with PD compared with relatives of controls (HR = 1.37), particularly in FDR of patients with PD with onset age ≤66 years (HR = 1.73). The results of this study showed that the incidences of different degrees of cognitive impairment were not significantly different between the siblings of EOP and LOP. This may result from the small sample of EOP, and the siblings of patients with EOP were relatively younger.

Siblings of patients with EOP have more NMS and are presumed to be at a higher risk of prodromal PD than those of patients with LOP. The reason for this may be related to the shared genetic factors and early life environment. Several studies have supported that the EOP are more associated with genetic factors. Researchers used to demonstrate the influence of genetic factors on disease by the epidemiological studies of twins. In 1999, an etiologic study on PD in twins found that the genetic component was not apparent when PD began after an age of 50 years; however, genetic factors had a major role when PD began at an age of ≤50 years [20]. In addition, a meta-analysis of the risk of PD in Asian populations showed that SNPs (rs3758549) were more common in patients with EOP [21]. A case-control study from Taiwan revealed that glucocerebrosidase (GBA) gene mutations were more associated with the risk of EOP [22].

Multifactorial risks result in the development of PD. In addition to genetic factors, special environment exposures are also confirmed as the risk factors for PD. Rajput et al. [23] reported in 1986 a relationship between early survival environment and EOP. They evaluated the childhood environment in 21 PD patients with onset age ≤40 years. Of these, 19 patients had been living in rural areas before the age of 15 years and 20 used exclusively well water in the first 15 years of life. Finally, they speculated that the rural environments and drinking well water in childhood might be potential risk factors for EOP. De Carvalho Aguiar et al. [24] investigated the mutations in PARK2 and PARK8 and environmental factors for EOP. They revealed that 18% of patients with EOP had mutations and 32% of them had PD family history. They also observed a positive correlation between EOP and long-term well water drinking before adulthood. They concluded that the onset of EOP was caused by interactions between the PARK gene and early environmental factors.

Studies have shown that NTDP had a higher prevalence of NMS compared with TDP [15, 25, 26]. However, the present study found that siblings of patients with TDP had more NMS than siblings of patients with NTDP. It was speculated that the siblings of TDP might have a greater risk in the PD prodromal stage. In particular, RBD was one of the most representative symptoms of the prodromal PD and generally considered to be a sign of neurodegeneration [27]. Korchounov et al. [28] subdivided 366 patients with PD into tremor-dominant type (TDT), akinetic-rigid type (ART), and mixed type according to their motor symptoms. They revealed that a positive PD family history was significantly associated with TDT (OR = 5.7) and only patients with EOP in the ART group had more positive family history (OR = 7.8). Hence, they speculated an autosomal dominant mode of transmission in TDT.

Smoking and caffeine intake in adulthood have been associated with a low risk of PD [29, 30]. A large Italian multicenter case-control study revealed that smoking, alcohol intake, and caffeine intake were negatively correlated with the onset of PD, and this negative correlation was more pronounced in NTDP [31]. Another case-control study in Norwegian achieved similar results: smoking and alcohol use were associated with a lower risk of PD; however, this inverse association was not seen in TDP [32]. Therefore, it was speculated that the intake of caffeine and nicotine might not reduce the risk of TDP. The incidence of TDP might be more related to factors other than adulthood living environments, such as genetic factors and early life environment.

In summary, siblings of patients with EOP and sibling of patients with TDP had more NMS, presuming that they had a higher risk of neurodegeneration. This was probably because the shared genes and early survival environment were more likely to result in EOP and TDP. Whether these FDR have a greater possibility to progress into PD requires further long-term follow-up in the future. However, if multiple PD prodromal symptoms appear in the FDR of patients with EOP and FDR of patients with TDP, especially the siblings, a continuous follow-up assessment is recommended to obtain early diagnosis and start therapy in the early stages of the disease.

The present study has several limitations. First of all, the sample size was small, especially the number of siblings of patients with EOP. The statistical power of some parameters was significantly low. Second, RBDSQ was a self-screening questionnaire with relatively low sensitivity and specificity. Due to the limited conditions, RBD could not be diagnosed by polysomnography, which is an objective diagnosis standard. Third, lack of pesticides exposure in the baseline information. After all, the study has confirmed that pesticide exposure is one of the environmental factors resulting in the high risk of PD [33]. Fourth, a large number of siblings of patients with PD were not enrolled in the survey due to migration, illness, unwillingness, old age, lack of time, and other reasons. Some of them might have had cognitive impairments with difficult to communicate with others or had emotional problems without the desire to communicate. All these might have led to deviations in the results.

## Figures and Tables

**Table 1:** Baseline data of the FDR of patients with EOP and LOP.

	FDR of patients with EOP (*n*=36)	FDR of patients with LOP (*n*=220)	Comparison between the two groups	
			Test value	*P* value
Age (year, mean ± SD)	64.5 ± 7.2	66.3 ± 9.9	*t* = 254	*P*=0.31
Education (year, mean ± SD)	8.7 ± 3.6	7.2 ± 4.2	*t* = 253	**P=0.04**
Gender (F), *n* (%)	17 (47.2%)	96 (43.6%)	χ^2^ = 0.161	*P*=0.69
Smoking, *n* (%)	12 (33.3%)	108 (49.1%)	χ^2^ = 3.085	*P*=0.08
Drinking tea, *n* (%)	7 (19.4%)	62 (28.2%)	χ^2^ = 1.200	*P*=0.27
Drinking coffee, *n* (%)	3 (8.3%)	15 (6.8%)	χ^2^ = 0.109	*P*=0.74
Risk factors for vascular diseases, *n* (%)	11 (30.6%)	98 (44.5%)	χ^2^ = 2.477	*P*=0.12

Abbreviations: FDR, first-degree relatives; EOP, early-onset Parkinson's disease; F, female; LOP, late-onset Parkinson's disease; SD, standard deviation.

**Table 2:** Comparison of NMS between the FDR groups of EOP and LOP.

	FDR of patients with EOP (*n*=36)	FDR of patients with LOP (*n*=220)	Comparison between the two groups		Power
			OR (95% CI)	*P* value	
RLS, *n* (%)	8 (22.3%)	29 (13.2%)	2.15 (0.88–5.25)	0.094	0.27
RBD, *n* (%)	6 (16.7%)	26 (11.8%)	2.93 (0.96–8.92)	0.058	0.12
Daytime sleepiness, *n* (%)	5 (13.9%)	15 (6.8%)	3.40 (1.00–11.48)	**0.049**	0.27
Constipation, *n* (%)	4 (11.1%)	24 (10.9%)	1.55 (0.46–5.24)	0.484	0.05
Sleep disorders, *n* (%)	3 (8.3%)	37 (16.8%)	0.98 (0.28–3.42)	0.977	0.30
Cognitive impairment, *n* (%)	9 (25.0%)	58 (26.4%)	2.16 (0.80–5.84)	0.133	0.05
Possible MCI (MoCA 22–25), *n* (%)	9 (25.0%)	46 (20.9%)	2.57 (0.99–6.68)	0.053	0.08
Possible dementia (MoCA ≤ 21), *n* (%)	0	12 (5.5%)	1.06 (1.03–1.09)	0.227	0.74
Depression, *n* (%)	6(16.7%)	26(12.3%)	1.72 (0.62–4.77)	0.294	0.11
Mild depression (BDI-II 14–19), *n* (%)	1 (2.8%)	14 (6.4%)	0.37 (0.04–3.41)	0.414	0.17
Moderate to severe depression (BDI-II ≥20), *n* (%)	5 (13.9%)	12 (5.9%)	4.08 (1.12–14.92)	**0.033**	0.32
Anxiety, *n* (%)	16(44.4%)	49(22.2%)	4.22 (1.87–9.52)	0.001	0.76
Mild anxiety (SAS 50–59), *n* (%)	8 (22.2%)	39 (17.7%)	1.76 (0.70–4.40)	0.229	0.09
Moderate to severe anxiety (SAS ≥ 60), *n* (%)	8 (22.2%)	10 (4.5%)	9.37 (2.86–30.67)	**<0.001**	0.86

Abbreviations: NMS, nonmotor symptoms; FDR, first-degree relatives; CI, confidence interval; EOP, early-onset Parkinson's disease; LOP, late-onset Parkinson's disease; MCI, mild cognitive impairment; RBD, rapid eye movement sleep behavior disorder; RLS, restless legs syndrome; MoCA, Montreal cognitive assessment; BDI-II, Beck Depression Inventory-II; SAS, Self-Rating Anxiety Scale.

**Table 3:** Baseline data of the FDR of patients with TDP and NTDP.

	FDR of patients with TDP (*n*=128)	FDR of patients with NTDP (*n*=128)	Comparison between the two groups	
			Test value	*P* value
Age (year, mean ± SD)	64.6 ± 8.4	67.4 ± 10.5	*t* = 254	*P*=0.02
Education (year, mean ± SD)	7.3 ± 3.7	7.4 ± 4.5	*t* = 253	*P*=0.88
Gender (female), *n* (%)	60 (46.9%)	53 (41.4%)	χ^2^ = 0.78	*P*=0.38
Smoking, *n* (%)	57 (44.5%)	63 (49.2%)	χ^2^ = 0.57	*P*=0.45
Drinking tea, *n* (%)	29 (22.7%)	40 (31.3%)	χ^2^ = 2.40	*P*=0.16
Drinking coffee, *n* (%)	8 (6.3%)	10 (7.8%)	χ^2^ = 0.24	*P*=0.63
Risk factors for vascular diseases, *n* (%)	55 (43.0%)	54 (42.2%)	χ^2^ = 0.16	*P*=0.90

Abbreviations: FDR, first-degree relatives; TDP, tremor-dominant Parkinson's disease; NTDP, non-tremor-dominant Parkinson's disease; SD, standard deviation.

**Table 4:** Comparison of NMS between the FDR groups of TDP and NTDP.

	FDR of patients with TDP (*n*=128)	FDR of patients with NTDP (*n*=128)	Comparison between the two groups		Power
			OR (95% CI)	*P* value	
RLS, *n* (%)	21 (16.4%)	16 (12.5%)	1.50 (0.72–3.11)	0.280	0.14
RBD, *n* (%)	25 (19.5%)	7 (5.5%)	11.65 (3.82–35.54)	**<0.001**	0.94
Daytime sleepiness, *n* (%)	12 (9.4%)	8 (6.3%)	2.82 (0.97–8.17)	0.056	0.16
Constipation, *n* (%)	20 (15.6%)	8 (6.3%)	4.94 (1.85–13.21)	**0.001**	0.67
Sleep disorders, *n* (%)	23 (18.0%)	17 (13.3%)	4.51 (1.73–11.78)	**0.002**	0.18
Cognitive impairment, *n* (%)	58 (29.7%)	29 (22.7%)	3.55 (1.62–7.77)	**0.002**	0.25
Possible MCI (MoCA 22–25), *n* (%)	33 (25.8%)	22 (17.2%)	3.19 (1.51–6.74)	**0.002**	0.39
Possible dementia (MoCA ≤ 21), *n* (%)	5 (3.9%)	7 (5.5%)	2.64 (0.53–13.08)	0.235	0.10
Depression, *n* (%)	17 (13.3%)	16 (12.6)	1.16 (0.53–2.53)	0.717	0.05
Mild depression (BDI-II 14–19), *n* (%)	7 (5.5%)	8 (6.3%)	0.83 (0.28–2.48)	0.738	0.06
Moderate to severe depression (BDI-II ≥20), *n* (%)	10 (7.8%)	8 (6.3%)	1.64 (0.53–5.13)	0.392	0.08
Anxiety, *n* (%)	40 (31.2%)	25 (19.6%)	2.49 (1.32–4.71)	**0.005**	0.56
Mild anxiety (SAS 50–59), *n* (%)	30 (23.4%)	17 (13.3%)	2.52 (1.24–5.15)	**0.011**	0.55
Moderate to severe anxiety (SAS ≥ 60), *n* (%)	10 (7.8%)	8 (6.3%)	1.79 (0.62–5.13)	0.279	0.08

Abbreviations: NMS, nonmotor symptoms; FDR, first-degree relatives; CI, confidence interval; TDP, tremor-dominant Parkinson's disease; NTDP, non-tremor-dominant Parkinson's disease; MCI, mild cognitive impairment; RBD, rapid eye movement sleep behavior disorder; RLS, restless legs syndrome; MoCA, Montreal cognitive assessment; BDI-II, Beck Depression Inventory-II; SAS, Self-Rating Anxiety Scale.

## Data Availability

The data used to support the findings of this study are available from the corresponding author upon request.
